# Circulating HIV DNA Correlates With Neurocognitive Impairment in Older HIV-infected Adults on Suppressive ART

**DOI:** 10.1038/srep17094

**Published:** 2015-11-25

**Authors:** Michelli Faria de Oliveira, Ben Murrel, Josué Pérez-Santiago, Milenka Vargas, Ronald J. Ellis, Scott Letendre, Igor Grant, Davey M. Smith, Steven Paul Woods, Sara Gianella

**Affiliations:** 1 University of California San Diego, La Jolla, CA, USA; 2 HIV Neurobehavioral Research Center, San Diego, CA, USA; 3 Veterans Affairs San Diego Healthcare System, San Diego, CA, USA; 4 University of Houston, Houston, TX, USA

## Abstract

Older HIV-infected adults have a higher risk of neurocognitive impairment, but the underlying mechanisms are poorly understood. Here, we investigated the associations between levels of HIV DNA in peripheral blood, soluble markers of inflammation and cellular trafficking in blood and cerebrospinal fluid (CSF) and neurocognitive functioning among 18 younger (22–40 years) and 26 older (50–71 years) HIV-infected subjects, who were administered a comprehensive neurocognitive battery. Older HIV-infected individuals presented higher levels of inflammation in CSF and blood compared to younger individuals, but no difference was observed in HIV DNA levels. Among older participants, higher HIV DNA levels were significantly associated with more severe neurocognitive impairment (p = 0.005), particularly in the Executive Functions domain (p = 0.004). No association was observed between HIV DNA and neurocognition among younger individuals. Despite significantly increased inflammation observed in the older group, none of the inflammatory markers were associated with neurocognitive impairment among older HIV+ individuals (p > 0.05). Our study supports the involvement of peripheral HIV DNA reservoir in the pathogenesis of neurocognitive disorder during suppressive ART. Correlates of neurocognitive impairment might differ between younger and older adults, suggesting that future treatment and prevention strategies for HIV-associated neurocognitive disorders likely need to be tailored based on age.

A notable shift in the demographics of the HIV epidemics has been observed over the last decade, with individuals over 50 years of age expected to comprise more than 75% of the HIV-infected population in the United States by 2020^1^. As expected, a parallel increase in aging-related complications such as cardiovascular, liver and kidney disease has been observed, even when antiretroviral treatment (ART) is successful. Older HIV-infected adults also have a three-fold higher risk of developing neurocognitive impairment (NCI) compared to younger HIV-infected individuals^2^,^3^, and this health burden is likely to grow as this vulnerable population ages. Furthermore, older HIV-infected adults with NCI may be more likely to exhibit declines in critical aspects of everyday functioning, such as medication adherence^4^.

Although the advent of ART has drastically reduced the prevalence of more severe forms of NCI, the prevalence of milder forms (e.g., Asymptomatic Neurocognitive Impairment and Mild Neurocognitive Impairment) remains high, affecting up to half of all HIV-infected individuals^5^,^6^. In the ART-naive population, NCI has been associated with greater HIV RNA and DNA levels in plasma and cerebrospinal fluid (CSF), several inflammatory and monocyte activation markers, and lower current and nadir CD4 T-cell counts^7^,^8^,^9^,^10^,^11^,^12^,^13^,^14^,^15^, but these associations are not consistently found after viral suppression^16^,^17^. In subjects on suppressive ART, factors associated with NCI are mostly related to low CD4 nadir, demographics and comorbidities, like co-infections, cardiovascular disease and increased age^5^,^18^,^19^,^20^. Prior studies reported that a larger HIV DNA reservoir in peripheral blood was associated with more severe NCI^10^,^21^,^22^, and that this association was independent of HIV RNA viral load^11^. In the setting of virologic suppression, greater HIV DNA contributes to persistent immune activation in tissues, including the brain^23^. Such persistent immune activation in turn contributes to neurodegeneration and cognitive impairment^24^. These effects are likely to be cumulative and to differ based on the duration of HIV disease and age, as a consequence of multiple factors like immune senescence and altered blood-brain barrier permeability^25^. As such, it is important to identify factors specifically associated with neurocognitive impairment in older HIV individuals in the current era of suppressive ART. The primary goal of this study goal was to evaluate HIV proviral burden in circulating peripheral blood mononuclear cells as a potential correlate of neurocognitive impairment in relation to age.

## Results

### Demographic and clinical characteristics of age-stratified HIV-positive participants

The final HIV-infected cohort consisted of 40.9% younger (range: 22– 40 years, N = 18) and 59.1% older (range: 50–71 years, N = 26) individuals. All participants were on ART with suppressed HIV RNA levels in blood plasma, and 38 subjects (86.4%) also had suppressed HIV RNA levels in CSF. At the time of sample collection, 31 (70.5%) subjects were on a protease inhibitor (PI)-based ART and 13 (29.5%) subjects were on a combination regimen consisting of a nucleoside reverse transcriptase inhibitor (NRTI) with either a non-nucleoside reverse transcriptase inhibitor (NNRTI) or an integrase strand transfer inhibitor (INSTI). Demographic and clinical characteristics are summarized in Table 1. No significant differences between the younger and older groups were observed for most demographic and clinical characteristics, including mood and substance use disorders, (p > 0.10). However, as might be expected, older individuals presented with longer EDI (p < 0.0001) and longer lifetime exposure to ART (p = 0.009) than younger individuals.

### Effect of HIV infection on NC performance

**Figure 1:**
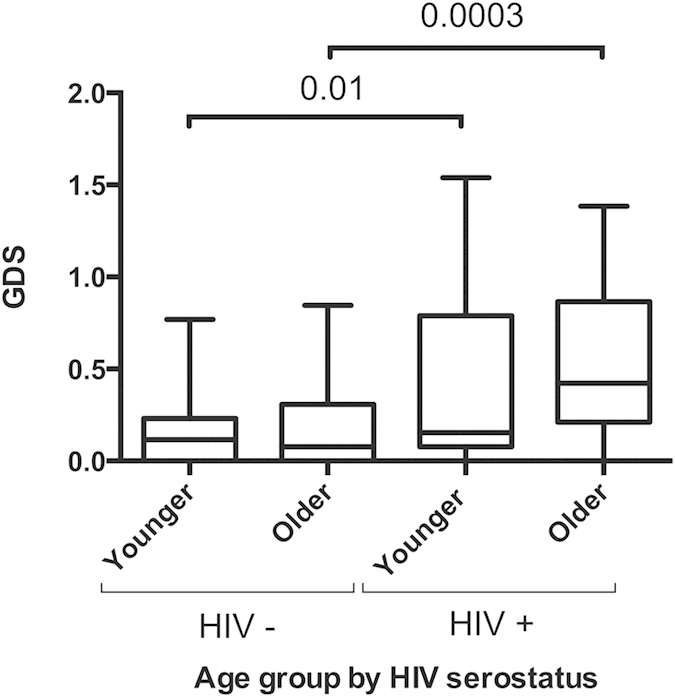
Effect of HIV serostatus on GDS. HIV infection presented significant effect on GDS levels in both older and younger groups. T-exact test p values are indicated.

To evaluate the effects of HIV infection on neurocognitive functioning, we included 46 younger and 35 older HIV-negative controls matched by age, sex and ethnic identity to their HIV infected counterparts (p > 0.1). Post-hoc comparisons showed a significant effect of HIV-serostatus on GDS levels in both older (p = 0.0003) and younger (p = 0.01) groups (Fig. 1; t-test), confirming that HIV-infection is associated with worse neurocognitive functioning independently of age.

### Differences in HIV DNA and markers of inflammation between age groups

Subsequently, we investigated any differences in HIV DNA and inflammatory markers levels in CSF and blood between age groups. HIV DNA levels in peripheral blood mononuclear cells (PBMC) were not significantly different between older and younger individuals (2.4 HIV DNA log_10_ per million CD4 T cells [interquartile range (IQR): 1.9–2.6] versus 2.5 [1.9–2.7], (p = 0.96, Table 2). When compared to younger individuals, older participants presented higher levels of sCD163 (p < 0.0001) and IL-8 (p = 0.03) in CSF (but not in blood plasma) and higher levels of IL-6 (p = 0.03) in blood plasma (but not in CSF). Older individuals also had higher levels of MCP-1 in both blood (p = 0.03) and CSF (p = 0.01) compared to younger subjects. After adjusting for EDI, only sCD163 levels in CSF and blood remained significantly higher among older individuals than in the younger group (p = 0.01 and p = 0.03; Table 2). To evaluate whether differences in cytokines levels between older and younger age groups were caused by local production or traffic between compartments, we estimated the CSF/serum albumin ration for each participant. No statistical difference was observed in the CSF/serum albumin ratios between the two age groups (p = 0.16), suggesting no significant differences in the blood brain barrier integrity between younger and older populations.

Additionally, we were only able to detect the cellular gene RPP30 in 13 (of 44) CSF cellular pellets (median: 16.8 cells; range: 0–3.160 cells) and HIV DNA in 2 CSF pellets - 1 from a younger subject with suppressed viral load in CSF, and 1 from an older subject with detectable HIV RNA in CSF. Among six subjects with detectable HIV RNA in CSF (142 [85.2–153.8] copies/mL), 2 were younger and 4 were older subjects and this was not statistically significant.

### HIV DNA, markers of inflammation and NCI

**Figure 2:**
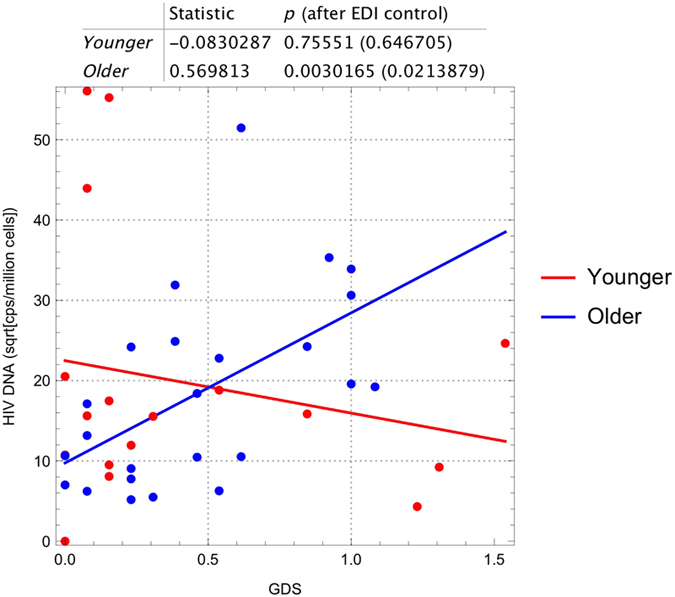
Association between neurocognitive function (GDS) and levels of HIV DNA by age group. Higher HIV DNA levels were associated with worse neurocognitive function in older, but not in younger, individuals. Regression analysis is indicated and repeated while controlling for EDI.

As part of our primary analysis, we evaluated whether HIV DNA levels in peripheral blood and other inflammatory or clinical markers were associated with worse NCI in each age group. Using an unadjusted regression model, we failed to find an association between HIV DNA levels and GDS among the younger group (r = −0.08, p > 0.5; Table 3). However, for older subjects, higher levels of HIV DNA were significantly associated with worse NCI (r = 0.57, p = 0.003) and this association remained significant after adjusting for EDI (p = 0.02; Table 3 and Fig. 2).

To assess whether the relationship between HIV DNA and GDS is reliably different between age groups, we regressed GDS against EDI, HIV DNA levels, age group, and the interaction between HIV DNA and age group. The interaction between HIV DNA levels and age group was significant at p = 0.02 (see supplementary Table S1 and S2). Among all other markers, only lower levels of sCD163 in CSF (r = −0.42, p = 0.03) were significantly associated with worse NCI among older individuals, but this association was not significant after adjusting for EDI (Table 3). No associations were observed between the other markers and NCI in any age group (p > 0.05), including current and nadir CD4 cell count.

### Subdomain analysis

**Figure 3:**
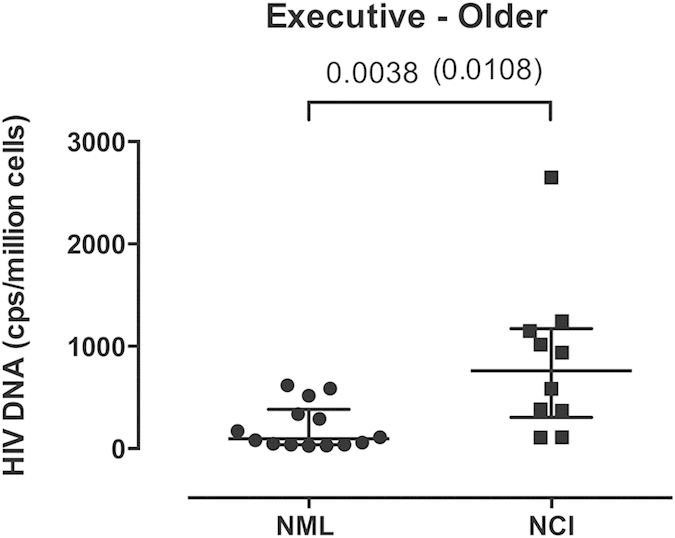
Levels of HIV DNA and the Executive Functions domain in older adults. Comparison of levels of HIV DNA between individuals with normal functioning and NCI in the Executive domain among older adults. Two-sided Mann-Whitney test p values are indicated. Analysis was repeated for while controlling for EDI (in brackets).

Lastly, we investigated whether the observed association between HIV DNA and global NCI was being driven by specific cognitive domains. In older subjects, higher levels of HIV DNA were significantly associated with worse Executive Functions (p = 0.004), which remained significant after adjusted analysis for EDI (p = 0.01; Fig. 3). No differences were found for any of the other tested domains (all p > 0.10).

## Discussion

The significance of different mediators of HIV-associated NCI may differ across the age spectrum and, to date, studies have not described a consensus marker focused on older patients. Determining mediators and biomarkers involved in the development of HIV-associated NCI, particularly among older adults taking suppressive ART, is essential for the design of future treatment and prevention strategies. To address this gap, our study was designed to investigate biomarkers specifically associated with NCI among older and younger HIV-infected adults.

In a first step, we compared neurocognitive functioning in older and younger HIV-infected and matched HIV-uninfected adults. We did observe a significant effect of HIV infection on NCI in both younger and older HIV infected groups compared to HIV negative age-matched controls, demonstrating that HIV infection can affect NC functioning independently of age.

Second, we observed that older HIV-infected individuals present higher levels of inflammatory markers in blood and CSF (in particular, sCD163, which is a marker of monocyte activation) compared to the younger individuals, but have similar albumin CSF/serum ratios. This suggests that the higher levels of monocyte activation observed in older subjects might be a consequence of a local inflammatory process in both compartments rather than a disruption of the brain blood barrier. Interestingly, none of these inflammatory markers were associated with worse NCI in the older population, which is in line with previous reports suggesting that inflammation might play a less central role in the development of NCI during suppressive ART^26^.

On the other hand, we found that older adults with higher levels of HIV DNA presented more severe NCI, particularly in the domain of executive functions (e.g., cognitive flexibility, planning). This effect was not evident in the younger HIV-infected population, despite similar levels of HIV DNA, neurocognitive functioning and cognitive reserve (i.e. years of education and verbal IQ) between age groups. This difference could be a consequence of enhanced transmigration of activated HIV-infected cells through the blood brain barrier, resulting in CNS inflammation, oxidative stress, and neuronal injury^27^,^28^, and deserves further investigation.

Levels of HIV DNA in peripheral blood have previously been associated with NCI^10^,^21^,^22^. In these studies, which included subjects from an Aging cohort in Hawaii, individuals with HIV–associated dementia presented higher levels of HIV DNA in PBMC than individuals with normal cognition, including a subset of 13 individuals with undetectable viral load^11^. Interestingly, no difference was noted between mildly impaired individuals and individuals with normal cognition^21^. Also, in the Hawaiian studies, levels of HIV DNA were significantly associated with cognitive impairment in all domains, except visuospatial ability^21^,^22^. Our data demonstrated a specific association between HIV DNA levels and the domain of executive functions, which is potentially important because executive dysfunction is increasingly prevalent during the ART era, among HIV+ individuals with well-managed disease (e.g., Heaton *et al.*, 2011). Executive dysfunction is also a robust predictor of poor everyday functioning outcomes (see Blackstone *et al.*, in press). In contrast with the previously published studies, our cohort includes only cases of non-severe NCI and subjects with normal cognition on suppressive ART, which is more relevant to the current HIV epidemics in the US; moreover, the main strength of our analyses is the design based on age categories, which is unique and relevant considering the ongoing changes in the HIV epidemiology characteristics.

Furthermore, in our study, we used the highly sensitive and precise digital droplet PCR (ddPCR) technology, which is particularly suitable for detection of low levels of HIV DNA, as expected to be the case in virally suppressed subjects.

Our study has several limitations. First, the small sample size and the cross-sectional design limited our ability to perform more detailed analyses on subgroups and longitudinally. For example, the lack of association between nadir CD4 count and NCI (previously reported by our group) could be a consequence of the limited sample size. Additionally, the observational study design did not allow inferring causality from the observed associations. Second, we normalized the HIV DNA levels based on the percentage of CD4 positive cells, despite the fact that some HIV DNA could have originated from non-CD4 expressing cells (particularly macrophages). Unfortunately, as a consequence of our retrospective study design, we were not able to measure HIV DNA levels in the monocyte population. Similarly, we were not able to measure HIV DNA levels using stored CSF cellular pellets, due the low number of cells in the majority of samples. Future studies should obtain freshly collected samples and possibly larger volumes of CSF in order to investigate the role of HIV-infected monocytes in the CNS and, ideally, in brain tissue from autopsy.

In summary, our study found that higher levels of peripheral CD4-associated HIV DNA was the main predictor of NCI among older HIV-infected adults on suppressive ART, and that this association was driven by a deficit in the Executive Functions domain. These findings add to emerging evidence that the correlates of NCI differ in older and younger HIV infected adults and suggest that future treatment and prevention strategies for HIV-associated neurocognitive disorders may need to be tailored on the basis of age. Strategies to reduce the HIV DNA reservoir will likely positively affect neurocognitive functioning in the HIV-infected population. For example, early initiation of ART might be particularly important for older individuals to limit CNS damage and prevent development of NCI. Future studies are needed to confirm our findings in a larger cohort, and to investigate possible mechanisms underlying this association.

## Methods

### Ethics Statement

All adult subjects provided their written informed consent. No children were included in this study. The study was carried out in accordance with the approved guidelines of the Office of Human Research Protections Program of the University of California, San Diego.

### Study population and sample

This study evaluated 44 HIV-positive and 81 HIV-negative subjects retrospectively selected from the HIV Neurobehavioral Research Center (HNRC)’s Prospective Memory cohort^29^. The parent study enrolled a total of 212 HIV-infected participants (without restrictions on ART status) between May 2008 and February 2013, and was designed to investigate the combined effects of age (in groups ≤40 and ≥50 years old) and HIV disease on NCI. HIV-infected participants on ART with suppressed HIV RNA levels in blood (<50 copies/μL of plasma) and with available paired blood and CSF samples qualified for this study. Blood CD4 + T lymphocyte subsets were measured by flow-cytometry (CLIA certified local laboratories), and HIV RNA levels were quantified by the Amplicor HIV Monitor Test (Roche Molecular Systems Inc.). HIV negative controls were matched by age, sex and ethnic identity to their HIV infected counterparts.

### Neurocognitive assessment

NCI was assessed using a standardized clinical battery of seven abilities areas consistent with Frascati recommendations for neuroAIDS research^30^. Analyses were based on the neurocognitive domains of Motor Skills, Executive Functions, Speed of Information Processing, Learning, Memory (Delayed Recall), and Attention and Working Memory (see Doyle *et al.* 2013^31^) for details on the specific test battery used). Raw scores were converted to demographically adjusted T-scores, which, in turn, were scaled to deficit scores ranging from 0 (normal, T>39) to 5 (severely impaired, T<20). Individual scores were averaged by domain, and overall NCI was summarized using the well-validated global deficit score (GDS), and parameterized either as a continuous variable, or as a binary variable (impaired or unimpaired), using the standard cutoff of 0.5^32^.

### HIV DNA from peripheral blood mononuclear cells (PBMC) and CSF

Genomic DNA was extracted from PBMC using QIAamp DNA Mini Kit (Qiagen) per manufacturer's protocol. Direct lysis was used on CSF cell pellets as previously described^33^. HIV DNA levels were assessed by digital droplet PCR (ddPCR). The human gene ribonuclease P (RPP30) is used in the ddPCR to normalize the number of HIV copies by the number of host cells and the results are reported as the number of copies of HIV DNA per 10^6^ million CD4 cells, as previously described^34^.

### Monocyte activation and inflammatory marker levels

We selected monocyte and macrophage activation markers, as well as specific inflammatory markers that have previously been associated with NCI in ART-naïve individuals^35^,^36^,^37^. Levels of selected monocyte activation and inflammatory markers were measured in CSF and blood plasma by enzyme-linked immunosorbent assay (ELISA): sCD163 (Trillium Diagnostics, Brewer, ME, USA); or by electrochemiluminescence multiplex assay: MCP-1, IL-8, IL-6 and TNF-α, (Meso Scale Diagnostics, Rockville, MD, USA), according to the manufacturer’s procedures.

### Albumin levels in blood plasma and CSF

Albumin levels in blood and CSF were measured by clinical assessment by ARUP Laboratories, Salt Lake City, UT, USA. The CSF/plasma albumin ratio was calculated as [CSF albumin (mg/dL)/serum albumin (mg/dL)] and was used to estimate the permeability of the blood brain barrier (BBB) as previously described^38^.

### Statistical analysis

Statistical analysis was performed using R and Mathematica statistical software. HIV DNA levels were log transformed and immunological covariates were square-root transformed to improve normality. Statistical differences between groups were evaluated using non-parametric tests (Mann-Whitney or Fisher Exact test). Associations between HIV DNA (primary hypothesis), and selected immunological markers and GDS were stratified by age group. Spearman correlations were used to assess the pairwise correlations between these markers and GDS, and, since EDI was significantly different between groups and could potentially affect GDS, associations were further examined after controlling for EDI in multivariate regression analysis. Finally, multivariate regression analysis was performed, with GDS as the outcome variable, and HIV DNA levels, age group, EDI and the interaction between HIV DNA level and age group as predictors.

## Additional Information

**How to cite this article**: Oliveira, M. F. *et al.* Circulating HIV DNA Correlates With Neurocognitive Impairment in Older HIV-infected Adults on Suppressive ART. *Sci. Rep.* **5**, 17094; doi: 10.1038/srep17094 (2015).

## Supplementary Material

Supplementary Information

## Figures and Tables

**Table 1:** Demographic and Disease Characteristics by age group.

Parameters n = 44	Younger group (N = 18)	Older group (N = 26)	p
Age (years), median [IQR]^a^	32 [28.5, 34.8]	57 [53, 62.5]	—
Education (years), median [IQR]^a^	13 [12, 13]	13.5 [12, 16]	0.12
Male, n [%]	13 [72.2]	20 [76.9]	1
Caucasian, n [%]	8 [44.4]	20 [76.9]	0.05
Global Deficit Score^a^	0.2 [0.1, 0.7]	0.4 [0.2, 0.8]	0.24
Psychiatric Diagnoses			
Any Affective Disorder, n [%]	13 [72.2]	17 [65.4]	0.74
Generalized Anxiety, n [%]	5 [27.8]	10 [38.5]	0.53
Current MDD, n [%]	4 [22.2]	2 [7.7]	0.20
Lifetime MDD, n [%]	13 [72.2]	16 [61.5]	0.53
LT Alcohol Dependence, n [%]	6 [33.3]	8 [30.8]	0.52
LT Alcohol Abuse, n [%]	3 [16.7]	6 [26.1]	0.71
LT Other Substance Abuse, n [%]	5 [27.8]	11 [47.9]	1
LT Other Substance Dependence, n [%]	8 [44.4]	13 [50]	0.77
EDI (years)^a^	8.4 [2.6, 12.6]	17.4 [15.2, 21]	**<0.0001**
Current CD4^a^	606 [494, 685]	722 [463.3, 901.3]	0.48
Nadir CD4^a^	216.5 [121.8, 321.8]	231 [92, 317.3]	0.73
Exposure ARVTs (years)^a^	3.9 [0.6, 8.8]	9.1 [6.0, 12.1]	**0.009**
CPE^a^	7 [7, 7.8]	7 [6, 8]	0.5

MDD = major depressive disorder; LT = lifetime; EDI = estimated duration of infection; CPE = CNS penetration effectiveness.

^a^Data shown as median [interquartile range].

Significant differences between younger and older p are determined by Mann-Whitney or Fisher test, as appropriate. Significant p values in bold.

**Table 2:** HIV DNA and cytokines levels between age group.

Parameters n = 44	Younger group (N = 18)	Older group (N = 26)	Mann-Whitney	Multivariate (EDI)
			p	p
HIV DNA (cps/million of cells; log_10_)	2.4 [1.9, 2.6]	2.5 [1.9, 2.7]	0.96	0.21
CSF				
sCD163 (ng/mL)	36.3 [24.6, 39.4]	61.3 [50.5, 69.1]	**<0.0001**	**0.01**
IL-6 (pg/mL)	1 [0.7, 1.2]	1 [0.7, 1.2]	0.25	
IL-8 (pg/mL)	40.1 [34, 47.4]	47.4 [42.7, 54.9]	**0.03**	0.21
MCP-1 (pg/mL)	328.2 [262.9, 413.3]	467 [327.3, 490.4]	**0.03**	0.06
Plasma				
sCD163 (ng/mL)	982.6 [786.8, 1441.8]	1501.9 [962.5, 1853.8]	0.07	**0.03**
IL-6 (pg/mL)	0.7 [0.6, 1.3]	0.9 [0.7, 1.2]	**0.03**	0.16
IL-8 (pg/mL)	5.1 [3.4, 7.6]	5.9 [4.8, 7.2]	0.17	
TNF-α (pg/mL)	1.6 [1.1, 2.1]	1.5 [1.2, 1.8]	0.73	
MCP-1 (pg/mL)	109.7 [85.2, 137]	131.9 [113.8, 157.3]	**0.01**	0.10

Data shown as median [interquartile range].

Significant differences between younger and older p are determined by Mann-Whitney. Significant p values in bold.

**Table 3:** Associations between GDS and the levels of HIV DNA and inflammatory markers by age group.

Predictor	Younger Group (n = 18)		Older Group (n = 18)		
	Spearman		Spearman		Multivariate (EDI)
	Statistic	p^a^	Statistic	p^a^	p^b^
HIV DNA	−0.08	0.76	0.57	0.003	**0.02**
Other possible predictors as described in the literature					
EDI	0.24	0.33	−0.41	0.04	
Nadir CD4	0.1	0.68	−0.09	0.64	
Current CD4	0.09	0.7	0.06	0.77	
CSF					
CD163 (ng/mL)	−0.24	0.35	−0.42	0.03	0.23
IL-6 (pg/mL)	−0.22	0.40	0.13	0.53	
IL-8 (pg/mL)	−0.08	0.75	0.14	0.51	
MCP-1 (pg/mL)	−0.40	0.13	−0.13	0.53	
Plasma					
CD163 (ng/mL)	−0.04	0.88	0.18	0.39	
IL-6 (pg/mL)	0.04	0.86	−0.30	0.14	
IL-8 (pg/mL)	0.09	0.70	0.23	0.26	
TNF-α (pg/mL)	−0.30	0.23	−0.21	0.30	
MCP-1 (pg/mL)	−0.13	0.60	−0.14	0.49	

EDI = estimated duration of infection.

^a^Significant associations p are determined by Spearman correlation.

^b^Multivariate analyses are determined after correction for EDI.
