# Corrigendum: Circulating HIV DNA Correlates With Neurocognitive Impairment in Older HIV-infected Adults on Suppressive ART

**DOI:** 10.1038/srep19387

**Published:** 2016-01-19

**Authors:** Michelli Faria de Oliveira, Ben Murrell, Josué Pérez-Santiago, Milenka Vargas, Ronald J. Ellis, Scott Letendre, Igor Grant, Davey M. Smith, Steven Paul Woods, Sara Gianella

Scientific Reports 5: Article number: 17094; 10.1038/srep17094 published online: 11252015; updated: 01192016.

The original version of this Article contained a typographical error in the spelling of the author Ben Murrell which was incorrectly given as Ben Murrel. This has now been corrected in the PDF and HTML versions of the Article.

