# Supplementary material for: Circulating HIV DNA Correlates With Neurocognitive Impairment in Older HIV-infected Adults on Suppressive ART
Source: Sci Rep. 2015 Nov 25;5:17094. doi: 10.1038/srep17094 (PMC4658529; doi:10.1038/srep17094)
Supplement: Supplementary Information [file srep17094-s1.doc]

**Circulating HIV DNA Correlates With Neurocognitive Impairment in Older HIV-infected Adults on Suppressive ART.**

Michelli Faria de Oliveira^1*^, Ben Murrel^1^, Josué Pérez-Santiago^1^, Milenka Vargas^1^, Ronald J. Ellis^2^, Scott Letendre^2^, Igor Grant^2^, Davey M. Smith^3^, Steven Paul Woods^4^, Sara Gianella^1*^.

*^1^University of California San Diego, La Jolla, CA, USA,*

*^2^HIV Neurobehavioral Research Center, San Diego, CA, USA,*

*^3^ Veterans Affairs San Diego Healthcare System, San Diego, CA, USA*

*^4^University of Houston, Houston, TX, USA.*

***Corresponding author and request for reprints:**

Michelli Faria de Oliveira, Ph.D.

University of California San Diego

9500 Gilman Drive MC 0679

La Jolla, CA 92093-0679, USA

Tel: (858) 552-8585 #2620

Fax: (858) 552-7445

E-mail: [mfariadeoliveira@ucsd.edu](mailto:joperez@ucsd.edu)

***Alternative corresponding author:**

Sara Gianella, M.D.

University of California San Diego

9500 Gilman Drive MC 0679

La Jolla, CA 92093-0679, USA

Tel: (858) 552-8585 #2673

Fax: (858) 552-7445

E-mail: [gianella@ucsd.edu](mailto:srmehta@ucsd.edu)

| Table S1. Explaining GDS with HIV DNA, Age group, and their interaction, while controlling for EDI. | | | | | |
| --- | --- | --- | --- | --- | --- |
|  | Estimate | Std. Error | t Value | P value | p<0.05 |
| (Intercept) | 0.230024 | 0.291933 | 0.788 | 0.4359 |  |
| Sqrt (HIV DNA) | 0.015565 | 0.007328 | 2.124 | 0.0406 | * |
| Age Group | 0.327439 | 0.241749 | 1.354 | 0.184 |  |
| EDI | -0.002826 | 0.012622 | -0.224 | 0.8241 |  |
| Sqrt (HIV DNA): Age Group | -0.022131 | 0.009356 | -2.365 | 0.0235 | * |

| Table S2. Effect Sizes: Modeling GDS |  |
| --- | --- |
| Multiple R^2^ | 0.152 |
| Zero order R^2^ Age Group | 0.007 |
| Zero order R^2^ sqrt(HIV DNA) | 0.01 |
| Zero order R^2^ EDI | 0.003 |
| sr^2^ for sqrt(HIV DNA):Age Group | 0.132 |
| Multiple R^2^ quantifies the variance explained by the entire model.  Zero order R^2^ quantify the raw effect of those variables, when no other variables are considered.  sr^2^ is the squared semi-partial correlation, which quantifies the change in explained variance when the sqrt(HIV DNA):Age Group interaction is added to the model, which reflects the variance uniquely explained by this term. | |
